# Precision Treatment of Colon Cancer Using Doxorubicin-Loaded Metal–Organic-Framework-Coated Magnetic Nanoparticles

**DOI:** 10.1021/acsami.4c08602

**Published:** 2024-09-03

**Authors:** Honglin Jiang, Qing Bao, Tao Yang, Mingying Yang, Chuanbin Mao

**Affiliations:** † School of Materials Science & Engineering, Zhejiang University, Hangzhou, Zhejiang 310027, China; ‡ Key Laboratory of Silkworm and Bee Resource Utilization and Innovation of Zhejiang Province, Institute of Applied Bioresource Research, College of Animal Science, Zhejiang University, Hangzhou, Zhejiang 310058, China; § Department of Biomedical Engineering, The Chinese University of Hong Kong, Shatin, Hong Kong SAR 999077, China

**Keywords:** metal−organic frameworks (MOFs), magnetic targeting, acid responsiveness, colon cancer, cardiotoxicity

## Abstract

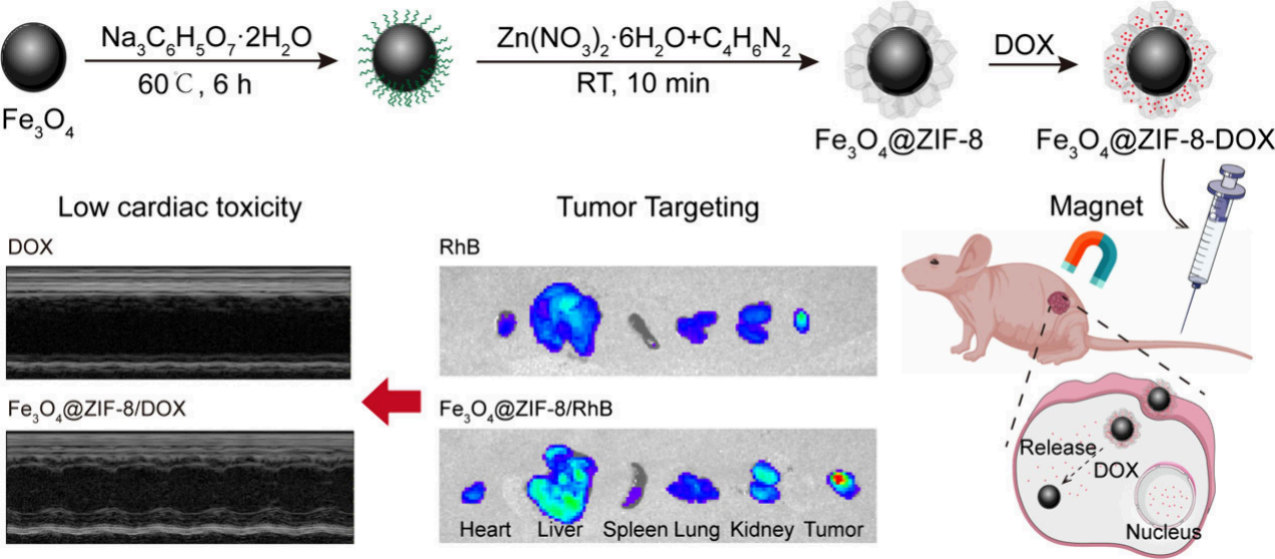

Due to the limited efficacy and evident side effects of traditional chemotherapy drugs attributed to their lack of specificity and selectivity, novel strategies are essential for improving cancer treatment outcomes. Here, we successfully engineered Fe_3_O_4_ magnetic nanoparticles coated with zeolitic imidazolate framework-8 (ZIF-8). The resulting nanocomposite (Fe_3_O_4_@ZIF-8) demonstrates efficient adsorption of a substantial amount of doxorubicin (DOX) due to the porous nature of ZIF-8. The drug-loaded nanoparticles, Fe_3_O_4_@ZIF-8/DOX, exhibit significant accumulation at the tumor site in SW620 colon-cancer-bearing mice when guided by an external magnetic field. Within the acidic microenvironment of the tumor, the ZIF-8 framework collapses, releasing DOX and effectively inducing tumor cell death, thereby inhibiting cancer progression while not causing undesired side effects, as confirmed by a variety of in vitro and in vivo characterizations. In comparison to free DOX, Fe_3_O_4_@ZIF-8/DOX nanoparticles show superior efficacy in colon cancer treatment. Our findings suggest that Fe_3_O_4_@ZIF-8 holds promise as a carrier for small-molecule drug adsorption and its ferromagnetic properties provide drug targeting capabilities, thereby enhancing therapeutic effects on tumors at the same drug dosage. With excellent biocompatibility, Fe_3_O_4_@ZIF-8 demonstrates potential as a drug carrier in targeted cancer chemotherapy. Our work suggests that a combination of magnetic targeting and acid-responsiveness holds great promise for advancing targeted cancer therapy in precision nanomedicine.

## 1 Introduction

Cancer poses a significant global health challenge, necessitating continuous efforts in the fields of medicine for its treatment and research.^1^ Chemotherapy stands out as a crucial method to combat cancer, effectively impeding the growth and spread of cancer cells.^2^ However, its lack of specificity for cancer cells often results in harm to normal cells, leading to notable side effects for patients.^3^ Additionally, the nontargeted nature of chemotherapy drugs limits their utilization and hampers therapeutic outcomes.^4,5^ Therefore, there is an urgent need to develop treatment approaches that are targeted and specific, with the aim to reduce side effects, improve treatment effectiveness, and enhance the quality of life for patients.

Thanks to the Enhanced Permeability and Retention (EPR) effect, nanoparticles are known for their increased propensity to accumulate within tumor tissues, making them an ideal drug delivery carrier.^6,7^ Fe_3_O_4_ nanoparticles have gained widespread acceptance in biomedical and clinical research owing to their strong magnetic responsiveness and minimal biotoxicity.^8,9^ Silica dioxide (SiO_2_) is a commonly used material for surface modification of Fe_3_O_4_ nanoparticles, which can be prepared with a porous structure for drug or gene loading.^10^ Porous silica-coated Fe_3_O_4_ nanoparticles, as drug delivery systems with magnetic targeting, have been extensively studied for cancer therapy.^11−13^ While they can target tumor tissue sites when exposed to an external magnetic field, this delivery method lacks control and selectivity in drug release.

Metal–organic frameworks (MOFs)^14,15^ are crystalline materials known for their precisely ordered structures.^16,17^ Among them, zeolitic imidazolate framework-8 (ZIF-8), a prominent example, holds significant applications in drug delivery due to its expansive surface area and unique porous structure.^18^ The crystal structure of ZIF-8 is susceptible to disruption in acidic environments, leading to a loss of stability and porosity, thereby releasing the loaded drugs.^19^ Given the acidic microenvironment of tumors, ZIF-8 selectively releases more drugs at tumor tissue sites, achieving acid-responsive drug release. Although the modified ZIF-8^20,21^ or composite materials containing ZIF-8^19,22−24^ possess pH sensitivity, they lack tumor-targeting ability, resulting in limited therapeutic efficacy.

Although Fe_3_O_4_ magnetic nanoparticles and ZIF-8 are commonly used materials, the composite material formed by their combination has not been reported in the field of colon cancer treatment. Based on the respective advantages and limitations of Fe_3_O_4_ nanoparticles and ZIF-8, we have chosen to employ porous ZIF-8-coated magnetic nanoparticles as a novel drug delivery platform for delivering the conventional chemotherapy drug doxorubicin to tumor sites. Doxorubicin (DOX) finds extensive application as a chemotherapy agent for the management of diverse cancer.^25^

**Scheme 1:**
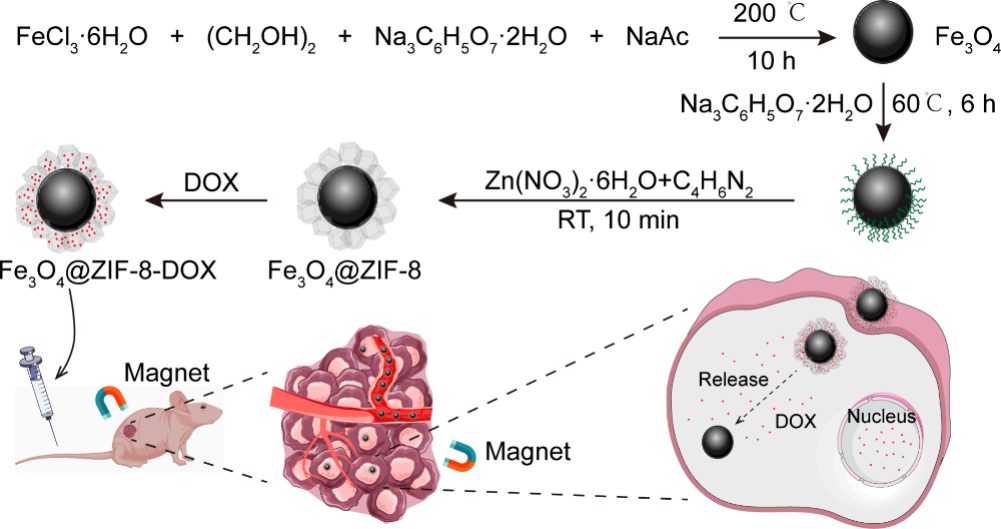
Diagram Outlining The Fabrication Process of Fe_3_O_4_@ZIF-8/DOX and Its Application in Magnetic-Targeted Therapy for SW620 Colon Cancer. Initially, Fe_3_O_4_ Nanoparticles Are Modified with a Layer of Citrate Groups, Capable of Capturing Metal Ions Such As Iron and Zinc Ions to Form Stable Chemical Bonds. Subsequently, ZIF-8 Nanoparticles Are Coated onto The Modified Fe_3_O_4_ Nanoparticles, With Its Porous Structure Capable of Adsorbing Drug Molecules. After the Produced Fe_3_O_4_@ZIF-8/DOX Nanoparticles Are Injected into Mice With SW620 Tumors, They Accumulate at The Tumor Site Under the Guidance of a Magnetic Field. Within The Acidic Tumor Microenvironment, The Porous Framework of ZIF-8 Collapses, Releasing DOX to Achieve Efficient Tumor-Killing Effects

Here, we have designed a drug delivery system utilizing ZIF-8-coated Fe_3_O_4_ magnetic nanoparticles. This system can load the traditional chemotherapy drug DOX, aggregate in tumor tissues guided by an external magnetic field, and achieve acid-responsive drug release within the acidic tumor microenvironment, enhancing the effectiveness of cancer treatment (Scheme 1). As both Fe_3_O_4_ nanoparticles and ZIF-8 exhibit good biocompatibility,^8,26^ the resulting composite material also demonstrates low cytotoxicity. Our innovative composite drug delivery system combines both targeting capabilities and acid responsiveness, offering a new paradigm for cancer therapy.

## 2 Results and Discussion

### Synthesis and Characterization of Fe_3_O_4_ Based Core–Shell Nanoparticles

**Figure 1:**
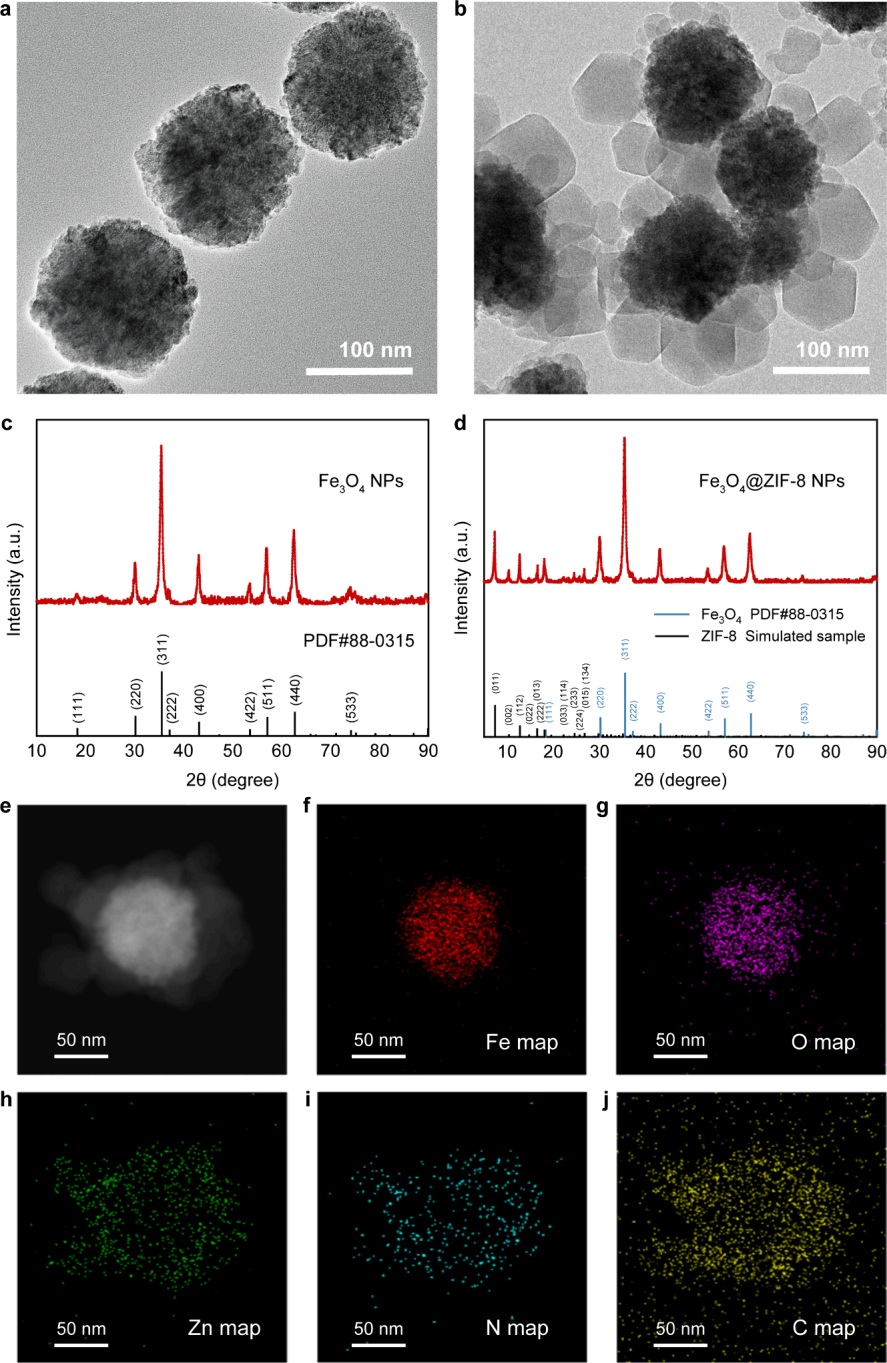
Synthesis and characterization of Fe_3_O_4_ based core–shell nanoparticles. a-b) TEM images of a) Fe_3_O_4_ and b) Fe_3_O_4_@ZIF-8. c-d) XRD spectrum of c) Fe_3_O_4_ and d) Fe_3_O_4_@ZIF-8. e) STEM image of Fe_3_O_4_@ZIF-8. f-j) Element mappings of Fe_3_O_4_@ZIF-8, including f) Fe, g) O, h) Zn, (i) N, and j) C.

In the creation of Fe_3_O_4_@ZIF-8 nanoparticles (Scheme 1), we first synthesized monodisperse Fe_3_O_4_ nanoparticles with a diameter of approximately 150 nm using a hydrothermal method,^27^ which exhibited a uniform spherical morphology (Figure 1a). Subsequently, we modified the surface of Fe_3_O_4_ nanoparticles with a layer of citrate groups, which can capture metal ions, such as iron and zinc ions, to form stable chemical bonds. This step laid the foundation for the surface coating of ZIF-8. Next, we achieved a uniform dispersion of the modified nanoparticles in a zinc nitrate solution and initiated a reaction with dimethylimidazole at room temperature. This process resulted in the formation of Fe_3_O_4_@ZIF-8 core–shell structured drug carriers. As shown in Figure 1b, we obtained composite nanoparticles with ZIF-8 thoroughly coated on the surface with a size of approximately 250 nm. The X-ray Diffraction (XRD) spectrum of Fe_3_O_4_ nanoparticles completely matched the standard powder diffraction file (PDF) of Fe_3_O_4_ (Figure 1c). The XRD pattern of the Fe_3_O_4_@ZIF-8 nanocomposite material also matched completely with the combination of the standard PDF of Fe_3_O_4_ and the simulated standard spectrum of ZIF-8 (Figure 1d), indicating that the composite nanoparticles we synthesized are consistent with the theoretical crystal structure. The core–shell architecture of Fe_3_O_4_@ZIF-8 was additionally corroborated through scanning transmission electron microscopy (STEM) (Figure 1e), along with elemental mapping of Fe, Zn, N, and C (Figure 1f-j). The images clearly illustrate the existence of Fe and O elements within the core as well as the presence of Zn, N, and C elements within the shell. These results collectively confirm the successful fabrication of core–shell structured nanoparticles composed of Fe_3_O_4_@ZIF-8 for drug delivery.

### Characterization of Drug-Loaded Nanoparticles

**Figure 2:**
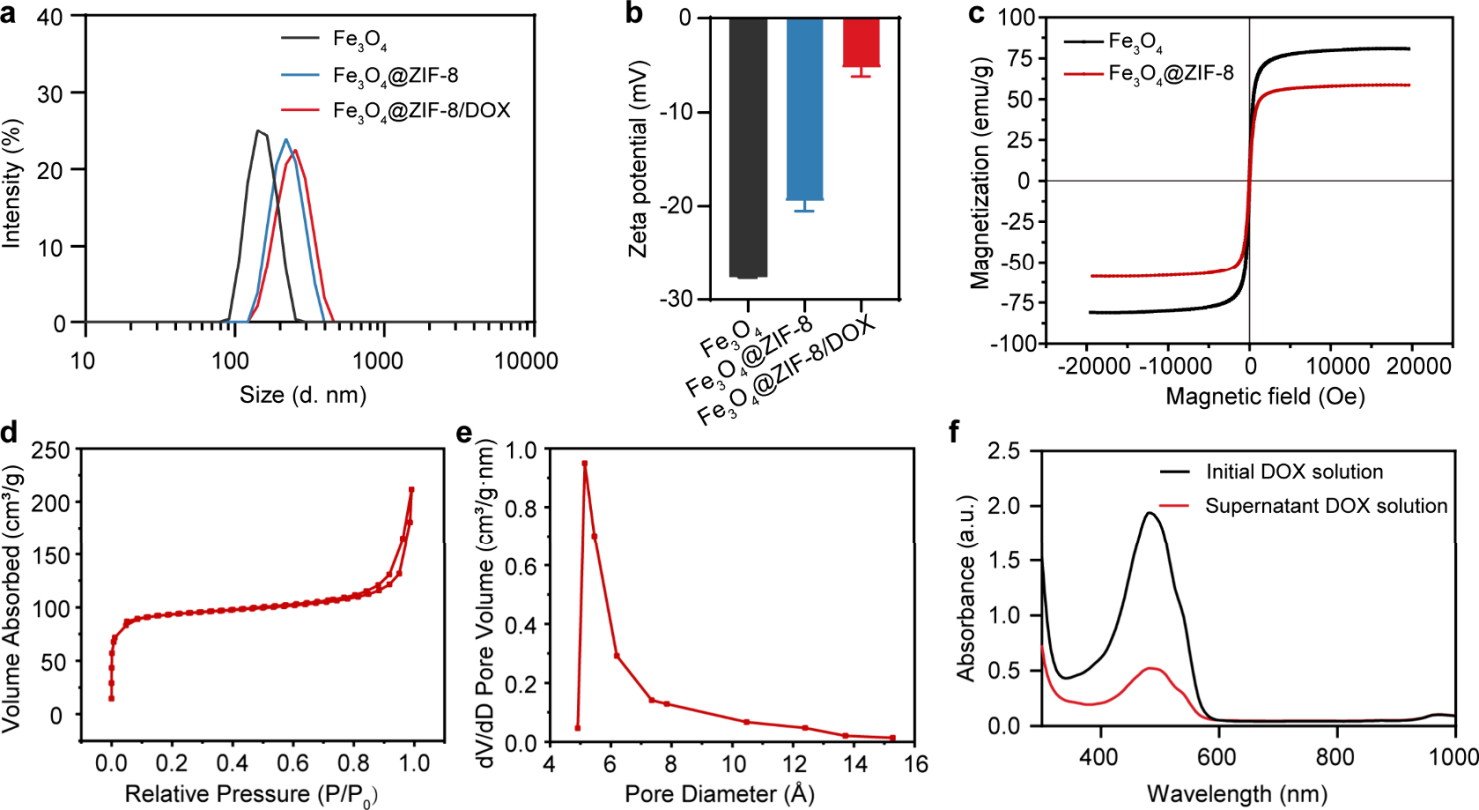
Characterization of the drug-loaded nanoparticles. a) Size and b) Zeta potential of Fe_3_O_4_, Fe_3_O_4_@ZIF-8 and Fe_3_O_4_@ZIF-8/DOX. (*n* = 3). c) VSM graphs for Fe_3_O_4_ and Fe_3_O_4_@ZIF-8. d) Nitrogen adsorption–desorption isotherm pattern for Fe_3_O_4_@ZIF-8. e) HK pore distribution of Fe_3_O_4_@ZIF-8. f) DOX encapsulation rate of Fe_3_O_4_@ZIF-8.

Before drug loading, we analyzed the pore structure of Fe_3_O_4_@ZIF-8 nanoparticles through Brunauer–Emmett–Teller (BET) testing. The nitrogen adsorption–desorption isotherm observed for Fe_3_O_4_@ZIF-8 nanoparticles exhibited typical features of a Type I isotherm, indicating that the nanoparticles have a microporous structure (Figure 2d). The pore size distribution confirmed the microporous nature of Fe_3_O_4_@ZIF-8 nanoparticles, with a median pore width of 5.62 nm (Figure 2e). Additionally, Fe_3_O_4_@ZIF-8 nanoparticles exhibited a substantial total pore volume (0.327263 cm^3^/g) and surface area (354.2326 m^2^/g), which are advantageous for drug loading. Coupled with their strong negative zeta potential (Figure 2b), Fe_3_O_4_@ZIF-8 nanoparticles are suitable for encapsulating cationic small-molecule drugs such as DOX. We further assessed the magnetic characteristics of both Fe_3_O_4_ and Fe_3_O_4_@ZIF-8 nanoparticles through the utilization of vibrating sample magnetometer (VSM) (Figure 2c). The saturation magnetization measured 81 emu/g for Fe_3_O_4_ nanoparticles and was slightly decreased to 59 emu/g for the composite nanoparticles (Fe_3_O_4_@ZIF-8), suggesting that the Fe_3_O_4_ nanoparticles still retain good ferromagnetic properties and responsiveness to an applied magnetic field after ZIF-8 encapsulation.

Various characterizations were conducted on drug-loaded Fe_3_O_4_@ZIF-8/DOX nanoparticles. Dynamic light scattering (DLS) analysis unveiled that the average particle size of Fe_3_O_4_ nanoparticles measured 154 nm, whereas the size of Fe_3_O_4_@ZIF-8 nanoparticles averaged 227 nm. These measurements align with the observations made in the TEM images (Figure 2a). After DOX was loaded into Fe_3_O_4_@ZIF-8/DOX, the average particle size of the Fe_3_O_4_@ZIF-8/DOX nanoparticles was increased to 251 nm. The loading of DOX transformed the nanoparticles from strongly to weakly negatively charged (Figure 2b). Upon incubation with Fe_3_O_4_@ZIF-8, the supernatant of the DOX solution displayed a notable reduction in absorbance at 483 nm, indicating that a substantial amount of DOX was adsorbed into the pores of Fe_3_O_4_@ZIF-8 (Figure 2f). Consequently, the encapsulation efficiency of DOX in the Fe_3_O_4_@ZIF-8 nanoparticles was as high as 77%.

### In Vitro Cancer Therapy of Fe_3_O_4_@ZIF-8/DOX Nanoparticles

**Figure 3:**
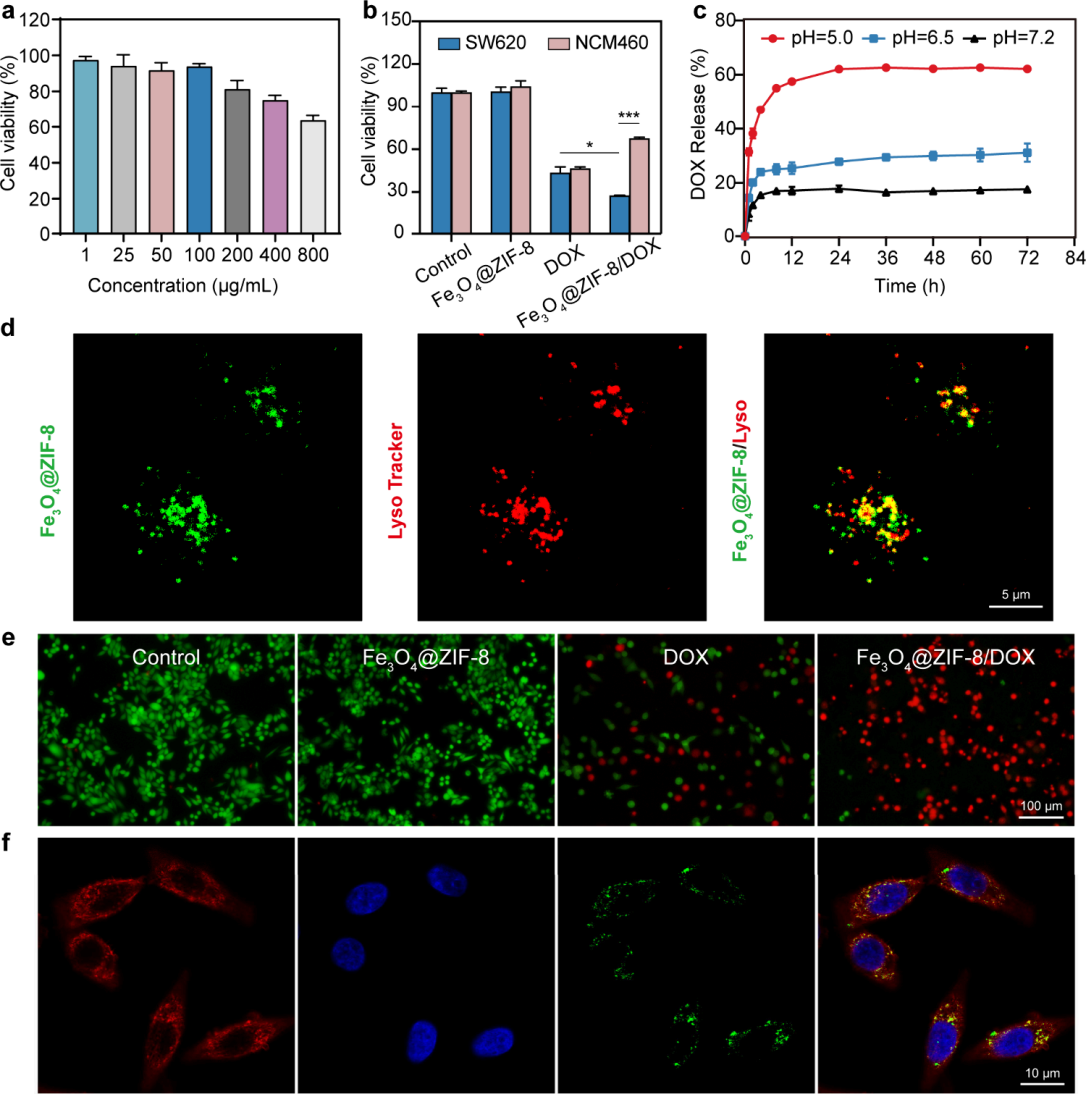
In vitro cancer therapy of Fe_3_O_4_@ZIF-8/DOX nanoparticles. a) SW620 cell viability after 48 h of incubation with varying concentrations of Fe_3_O_4_@ZIF-8. (*n* = 4). b) SW620 cell and NCM460 cell viability after 48 h of incubation with PBS, Fe_3_O_4_@ZIF-8, DOX and Fe_3_O_4_@ZIF-8/DOX. (*n* = 4). c) In vitro drug release curves under varying pH conditions (at pH values of 5.0, 6.5, and 7.2). (*n* = 3). d) Confocal fluorescence microscopy images of lysosome and FITC-labeled Fe_3_O_4_@ZIF-8. e) Live/dead staining of SW620 cells following 48 h of incubation with PBS, Fe_3_O_4_@ZIF-8, DOX or Fe_3_O_4_@ZIF-8/DOX. f) Confocal fluorescence microscopy images of SW620 cells following coincubation with FITC-labeled Fe_3_O_4_@ZIF-8. * *p* < 0.05, ** *p* < 0.01, *** *p* < 0.001.

We conducted in vitro experiments using SW620 colon cancer cells prior to the use of Fe_3_O_4_@ZIF-8/DOX nanoparticles in animal experiments. We proceeded to evaluate the cytotoxicity of Fe_3_O_4_@ZIF-8 nanoparticles using the Cell Counting Kit-8 (CCK-8) assay. SW620 cells were treated with varying concentrations of Fe_3_O_4_@ZIF-8 nanoparticles for 48 h, and the assessment of cell viability was conducted. The results in Figure 3a show that under the conditions of 100 μg/mL Fe_3_O_4_@ZIF-8 solution, the cell viability of SW620 remained above 90%, indicating the excellent biocompatibility of Fe_3_O_4_@ZIF-8. Therefore, we selected a concentration of 100 μg/mL of Fe_3_O_4_@ZIF-8 solution for subsequent in vitro SW620 cell experiments. To evaluate the efficacy of Fe_3_O_4_@ZIF-8/DOX in killing cancer cells, SW620 cells were separately cultured with Fe_3_O_4_@ZIF-8, free DOX, or Fe_3_O_4_@ZIF-8/DOX for 48 h. As depicted in Figure 3b, compared with free DOX, Fe_3_O_4_@ZIF-8/DOX was more effective in killing SW620 cancer cells. This could be attributed to the improved internalization of nanoparticles by the cells, facilitating the intracellular delivery of DOX, leading to improved cytotoxicity. Live/dead staining of SW620 cells further confirmed the superior cytotoxicity of Fe_3_O_4_@ZIF-8/DOX compared to that of free DOX at the same DOX concentration (Figure 3e). Nearly all SW620 cells treated with Fe_3_O_4_@ZIF-8/DOX were observed to be nonviable, displaying extensive red fluorescence. In contrast, in the group treated with free DOX, some green fluorescence-labeled surviving SW620 cells were observed. Additionally, SW620 cells treated with Fe_3_O_4_@ZIF-8 showed a growth status similar to the PBS control group, almost bearing no dead cells, indicating the good cell compatibility of Fe_3_O_4_@ZIF-8. These findings collectively validated the potential of Fe_3_O_4_@ZIF-8 nanoparticles as a drug delivery carrier, in good agreement with the CCK-8 results.

To further confirm the internalization of Fe_3_O_4_@ZIF-8 nanoparticles by SW620 cells, we labeled Fe_3_O_4_@ZIF-8 nanoparticles with fluorescein isothiocyanate (FITC) and visualized their cellular uptake by SW620 colon cancer cells in vitro using confocal fluorescence microscopy. As shown in Figure 3f, after SW620 cells were coincubated with FITC-labeled Fe_3_O_4_@ZIF-8 in the culture medium overnight, the FITC-labeled Fe_3_O_4_@ZIF-8 were efficiently taken up by SW620 cells and entered the cytoplasm of SW620 cells. Every cell exhibited green fluorescence. This outcome suggests that Fe_3_O_4_@ZIF-8 significantly enhances the DOX delivery efficiency in SW620 cancer cells, thereby increasing the utilization of the anticancer drug.

To verify the drug’s acid-responsive release, we assessed the drug release characteristics of Fe_3_O_4_@ZIF-8/DOX nanoparticles in buffer solutions with varying pH levels (Figure 3c). The findings indicated that after 72 h, the DOX release rates were 63% and 29% at pH 5.0 and 6.5, respectively. In contrast, the release rate at pH 7.2 under neutral conditions was only 18%. The pH sensitivity of Fe_3_O_4_@ZIF-8/DOX nanoparticles is due to the protonation of organic ligands in the ZIF-8 shell under acidic conditions, resulting in the rupture of Zn-imidazolium ion coordination bonds and subsequent decomposition of the ZIF-8 framework,^28^ thereby releasing DOX. The lysosomes of cancer cells have a lower pH value than those of normal cells,^29,30^ providing conditions for the acid-responsive DOX release from Fe_3_O_4_@ZIF-8/DOX nanoparticles. Besides, Fe_3_O_4_@ZIF-8/DOX exhibited lower cytotoxicity against healthy cells (NCM460 cells) than against SW620 colon cancer cells (Figure 3b). To further confirm the acid-responsive mechanism, we stained lysosomes in SW620 cells and found their colocalization with FITC-labeled Fe_3_O_4_@ZIF-8 (Figure 3d). These results confirmed that Fe_3_O_4_@ZIF-8/DOX nanoparticles exhibit acid responsiveness, allowing for the selective release of a substantial quantity of the drug within the acidic tumor microenvironment while maintaining structural stability in the neutral environment of normal cells.

### In Vivo Magnetic Targeted Therapy of Colon Cancer

Rhodamine B (RhB), alone or loaded into nanoparticles, was employed for in vivo imaging of SW620 tumor-bearing mice (Figure S1). After magnetic targeting for 1 h, Fe_3_O_4_@ZIF-8/RhB nanoparticles exhibited significantly stronger fluorescence at the tumor site than free RhB (Figure S1a-b). Moreover, the carrier Fe_3_O_4_@ZIF-8 nanoparticles prolonged the retention time of RhB in tumor tissue, whereas the fluorescence signal of free RhB rapidly diminished. At 12 h postinjection of free RhB and Fe_3_O_4_@ZIF-8/RhB nanoparticles, the fluorescence signals from isolated tumors and organs in SW620 tumor-bearing mice further confirmed the enhanced retention of RhB in various organs by nanoparticles, particularly in tumor tissues (Figure S1c-d), indicating the effect of magnetic targeting.

**Figure 4:**
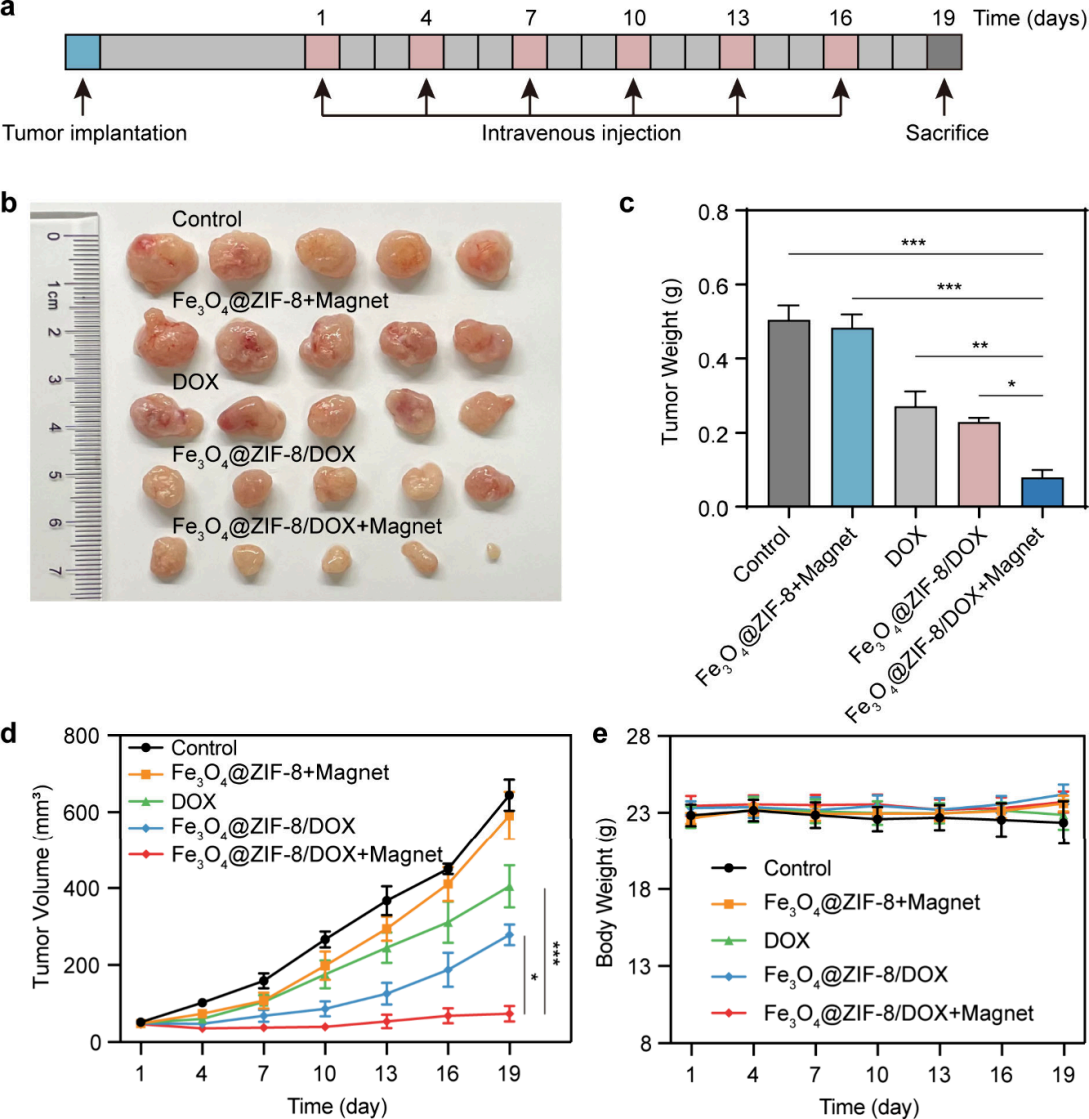
In vivo cancer therapy using Fe_3_O_4_@ZIF-8/DOX nanoparticles. (*n* = 5). a) Tumor therapy timeline. PBS, Fe_3_O_4_@ZIF-8, DOX, or Fe_3_O_4_@ZIF-8/DOX was given into SW620 tumor-bearing mice via tail vein every 3 days. b) Typical images and c) weight of tumors from SW620 tumor-bearing mice 19 days post various treatments. d) Tumor volume and e) body weight change in various treatment groups. * *p* < 0.05, ** *p* < 0.01, *** *p* < 0.001.

**Figure 5:**
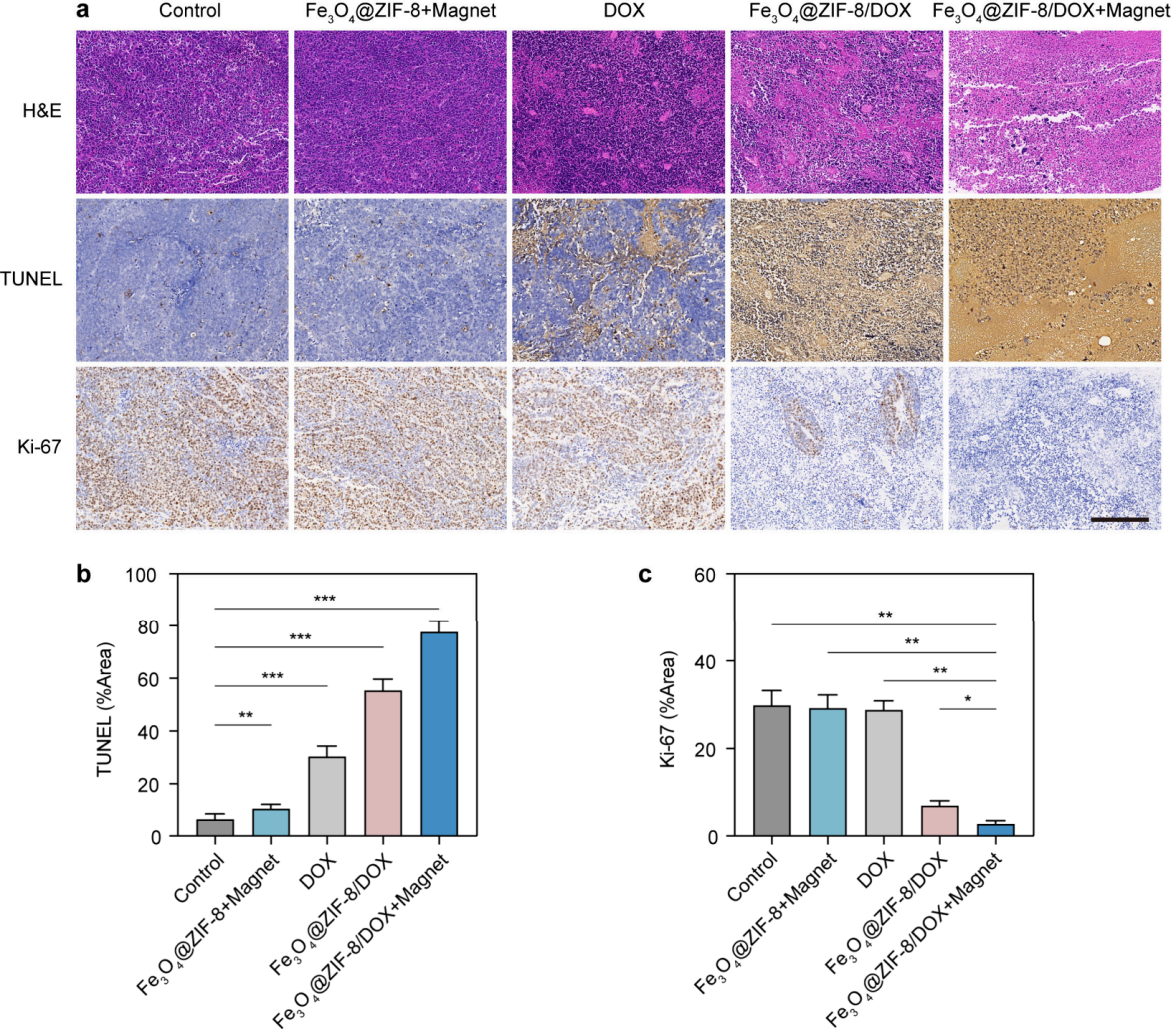
Stained images and semiquantitative analysis of tumor slices of nude mice. (*n* = 5). a) Schematic of H&E, TUNEL and *K*_i_-67 staining on SW620 tumor slices from nude mice subjected to different treatment regimens. The scale bar is 200 μm. b-c) Corresponding quantifications of immunohistochemically stained TUNEL (b) and *K*_i_-67 (c) in tumor. * *p* < 0.05, ** *p* < 0.01, *** *p* < 0.001.--.

Encouraged by the promising in vitro and in vivo imaging results, we conducted an in vivo study using mice bearing SW620 tumors to investigate the anticancer therapeutic effectiveness of the Fe_3_O_4_@ZIF-8/DOX nanoparticles. Once the tumor size reached 50 mm^3^, we intravenously administered the respective formulations to the tumor-bearing mice every 3 days (Figure 4a). Some groups received an additional 1 h application of an external magnetic field. Five groups were established: 1) Control (PBS), 2) Fe_3_O_4_@ZIF-8+Magnet, 3) free DOX, 4) Fe_3_O_4_@ZIF-8/DOX, and 5) Fe_3_O_4_@ZIF-8/DOX+Magnet. An external magnetic field was employed by affixing a magnet block to the tumor site of the mice immediately after drug injection and removed after a 1-h interval. After 19 days of treatment, tumors treated with Fe_3_O_4_@ZIF-8/DOX+Magnet were the smallest and lightest among all groups (Figure 4b-c). The tumor volume and body weight of SW620 tumor-bearing mice were recorded every 3 days. As illustrated in Figure 4d, tumors in mice treated with PBS or Fe_3_O_4_@ZIF-8+Magnet grew rapidly, indicating that Fe_3_O_4_@ZIF-8 nanoparticles alone lacked tumor-inhibitory capability. Free DOX had a very weak effect on inhibiting tumor growth. Fe_3_O_4_@ZIF-8/DOX exhibited more pronounced anticancer effects at an equivalent DOX concentration but still could not completely halt tumor growth. In contrast, tumors in the Fe_3_O_4_@ZIF-8/DOX+Magnet group showed significant growth inhibition, suggesting that the external magnetic field could confine Fe_3_O_4_@ZIF-8/DOX nanoparticles at the SW620 tumor site, enhancing their anticancer potency. Furthermore, the body weights of mice in five groups remained relatively stable throughout the treatment period, indicating that the drug formulations, concentrations, and dosing frequencies were biologically safe for the mice (Figure 4e). We performed H&E, TUNEL and *K*_i_-67 staining on tumor slices to evaluate the extent of damage, cell apoptosis, and cellular proliferation activity in tumor tissues under different treatments, respectively. As illustrated in Figure 5, the tumor slices from the experimental group exhibited the highest degree of damage, the most cell apoptosis, and the least active cellular proliferation, corroborating the excellent anticancer capabilities of Fe_3_O_4_@ZIF-8/DOX.

### Reduction of DOX-Induced Cardiotoxicity

**Figure 6:**
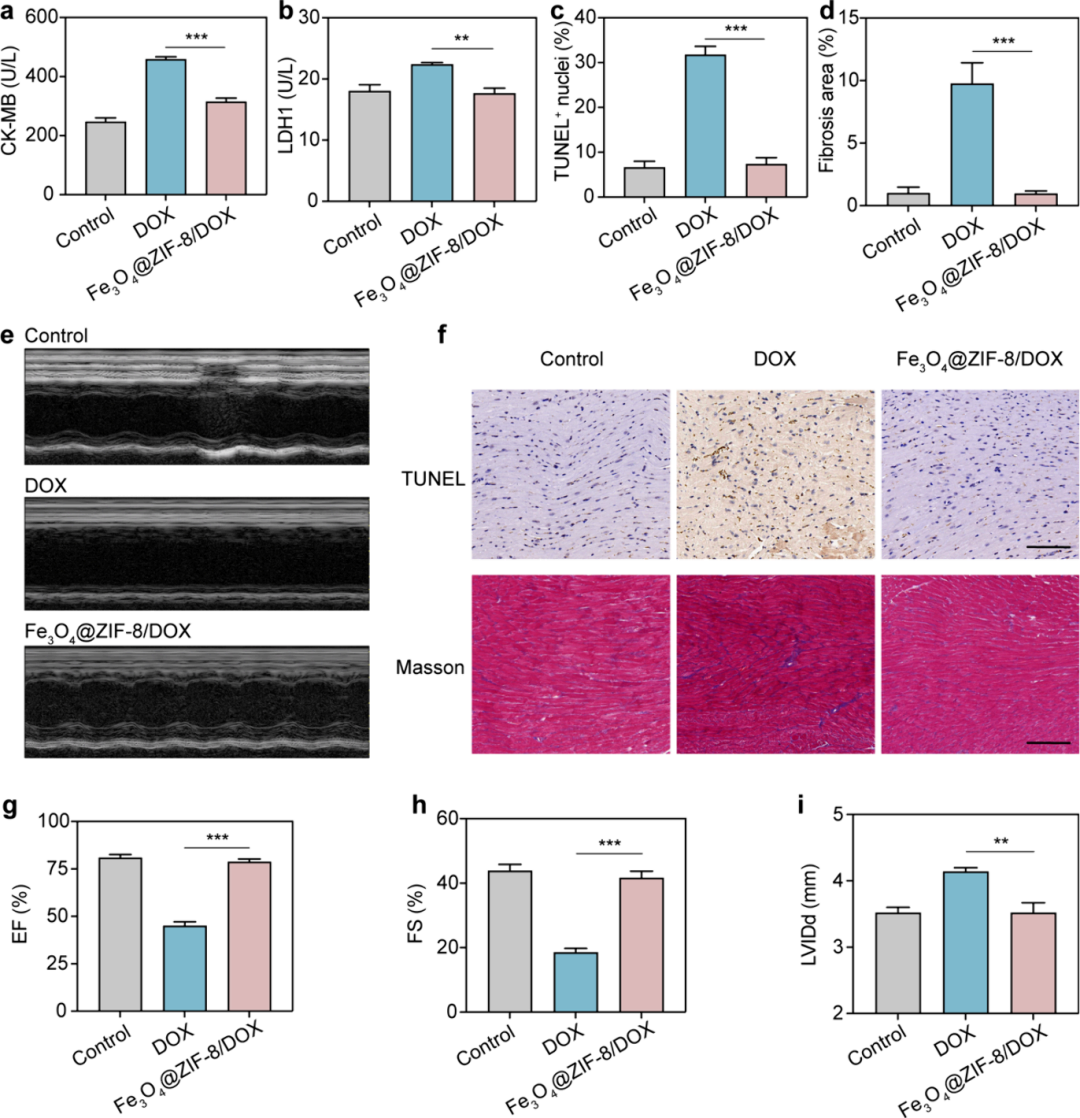
Fe_3_O_4_@ZIF-8/DOX nanoparticles reduced DOX-induced cardiotoxicity in mice. (*n* = 5). a) CK-MB and b) LDH1 levels associated with heart failure. Quantitative analyses of the c) TUNEL-positive cells and d) fibrotic area of heart slices. e) Representative M-mode echocardiographic images 19 days post various treatments. f) Schematic of TUNEL and Masson staining on heart slices from nude mice. The scale bar is 100 μm. Cardiac function was assessed by g) ejection fraction (EF), h) fractional shortening (FS) and (i) diastolic left ventricular internal diameter (LVIDd). ** *p* < 0.01, *** *p* < 0.001.

In both in vitro and in vivo studies, we confirmed the excellent tumor-killing effect of Fe_3_O_4_@ZIF-8/DOX nanoparticles. Indeed, the magnetic targeting not only significantly enhanced the efficacy of cancer therapy but also greatly reduced the side effects induced by DOX. Cardiotoxicity poses a major challenge in DOX-based cancer therapy and is manifested as myocardial damage, fibrosis, and decreased cardiac function.^31^ After 19 days of treatment, compared to mice treated with PBS, the DOX treatment increased the levels of biochemical parameters associated with heart failure, including LDH1 and CK-MB. Conversely, Fe_3_O_4_@ZIF-8/DOX nanoparticles with the same DOX dosage significantly reduced the levels of LDH1 and CK-MB, indicating lower myocardial damage (Figure 6a-b). To assess cardiac function, M-mode echocardiography of mice (Figure 6e) was performed and evaluated for the ejection fraction (EF), fractional shortening (FS), and diastolic left ventricular internal diameter (LVIDd). The Free DOX treatment group exhibited significantly decreased EF and FS, and significantly increased LVIDd (Figure 6g-i), suggesting the DOX-induced cardiac dysfunction in mice. In contrast, mice treated with Fe_3_O_4_@ZIF-8/DOX nanoparticles via magnetic targeting showed no signs of cardiac dysfunction, further confirming the ability of Fe_3_O_4_@ZIF-8/DOX nanoparticles to reduce cardiotoxicity. Furthermore, TUNEL and Masson staining of heart sections revealed that free DOX treatment increased apoptosis of cardiomyocytes and cardiac fibrosis, while Fe_3_O_4_@ZIF-8/DOX nanoparticles with the same DOX dosage could reduce DOX-induced cardiomyocyte apoptosis and cardiac fibrosis (Figure 6c-d, f). These results collectively demonstrate that using Fe_3_O_4_@ZIF-8/DOX nanoparticles for cancer magnetic targeting therapy can significantly mitigate the DOX-induced cardiotoxicity. The reduced cardiotoxicity is attributed to the magnetic targeting of Fe_3_O_4_@ZIF-8/DOX nanoparticles, which allows more drug-loaded nanoparticles to accumulate at the tumor site rather than in normal tissues. Additionally, the acid-responsive release capability of Fe_3_O_4_@ZIF-8/DOX nanoparticles prevents the release of DOX in the neutral environment of normal tissues.

### The Biocompatibility Evaluation

During the in vivo tumor treatment process, there were no notable instances of weight loss observed in any of the groups (Figure 4e), suggesting that Fe_3_O_4_@ZIF-8/DOX had no apparent side effects. To further validate their biocompatibility, we incubated Fe_3_O_4_@ZIF-8 nanoparticles at different concentrations with red blood cells for 4 h at 37 °C. The results indicated that even when using a high concentration of 6 mg/mL, the hemolysis rate remained below the threshold of 5% (ISO 10993–4:2017) (Figure S2), suggesting the feasibility of Fe_3_O_4_@ZIF-8/DOX nanoparticles entering the bloodstream via intravenous administration. We conducted histopathological analysis on key organs (heart, liver, spleen, lung, kidney) excised from the five sets of nude mice. The H&E staining images showed that the histological status of each organ was good (Figure S3), with no significant differences compared to the control group. This observation suggested that the therapeutic formulation did not result in tissue toxicity in nude mice. Furthermore, routine blood analysis (Figure S4a-f) and liver and kidney function analysis (Figure S5a-f) of SW620 tumor-bearing nude mice treated with Fe_3_O_4_@ZIF-8/DOX nanoparticles showed no significant differences compared to the control group. Taken together, these findings strongly indicate that Fe_3_O_4_@ZIF-8/DOX nanoparticles possess excellent biocompatibility and tissue safety, making them a promising therapeutic agent for combating SW620 colon cancer through magnetic targeting and acid-responsive drug release.

## 3 Conclusions

In summary, we synthesized Fe_3_O_4_ magnetic nanoparticles using a hydrothermal method and coated them with a ZIF-8 layer, creating a core–shell structured drug delivery carrier with dual functionalities of magnetic targeting and acid responsiveness. The resulting Fe_3_O_4_@ZIF-8 nanoparticles were effectively localized at the tumor sites under the influence of an external magnetic field. Moreover, in the acidic tumor microenvironment, the ZIF-8 structure underwent degradation, selectively releasing the anticancer drug DOX. Our Fe_3_O_4_@ZIF-8/DOX drug-loaded nanoparticles exhibited outstanding efficacy in killing tumor cells and demonstrated efficient acid-responsive drug release in vitro. Furthermore, they displayed significant tumor suppression in SW620 tumor-bearing mice in vivo. Importantly, Fe_3_O_4_@ZIF-8/DOX nanoparticles demonstrated excellent biocompatibility in vivo, presenting a promising avenue for the targeted treatment of colorectal cancer and other cancer types.

## 4 Methods

### Materials

All chemical compounds were utilized as obtained without requiring additional purification. Iron(III) chloride hexahydrate (FeCl_3_·6H_2_O), trisodium citrate dihydrate (Na_3_Cit·2H_2_O), sodium acetate (NaAc), zinc nitrate hexahydrate (Zn(NO_3_)_2_·6H_2_O), ethylene glycol and ethanol were purchased from HUSHI. 2-methylimidazol and doxorubicin hydrochloride were obtained from Sigma-Aldrich.

### Characterization

Morphology, STEM images, and element mapping were acquired using the JEOL JEM 2100F (Japanese). X-ray diffraction (XRD) spectra were recorded using Rigaku Ultima IV (Japanese). VSM curves were obtained from LakeShore7404. Particle size distribution and zeta potential were analyzed using Zetasizer Nano ZS90 (Malvern, n = 3). Nitrogen adsorption–desorption isotherm pattern was analyzed with a Micromeritics ASAP 2460 (USA).

### Synthesis of Fe_3_O_4_ Nanoparticles

0.27 g FeCl_3_·6H_2_O (0.05 M) and 0.2 g Na_3_Cit·2H_2_O were placed into 20 mL of ethylene glycol. After complete dissolution, 1.2 g of NaAc was introduced into the mixture, and stirring was continued for 30 min. Afterward, the mixture was transferred to a 50 mL reaction vessel and subjected to a reaction at 200 °C for 10 h.^27^ After being cooled to ambient temperature, the resultant precipitate was subjected to two rounds of washing with ethanol and deionized water. The Fe_3_O_4_ nanoparticles were gathered by centrifugation at 6000 rpm for a duration of 10 min.

### Surface Modification of Fe_3_O_4_ Nanoparticles

A solution was created by dissolving 29.41 g of Na_3_Cit·2H_2_O in 100 mL of deionized water. The previously synthesized Fe_3_O_4_ nanoparticles were dispersed in the solution with ultrasonication and mechanically stirred at 60 °C for 6 h. The precipitate was collected by magnetic attraction followed by a thorough wash with deionized water. The resulting modified Fe_3_O_4_ nanoparticles were then dissolved in 10 mL of deionized water.

### Synthesis of Fe_3_O_4_@ZIF-8 Nanoparticles

0.297 g amount of Zn(NO_3_)_2_·6H_2_O was dissolved in 10 mL of 50% ethanol to form a solution. One mL of citrate-modified Fe_3_O_4_ nanoparticles was introduced into the solution, which was then subjected to sonication for 15 min. The resulting mixture was transferred into 20 mL of 50% ethanol containing 5.736 g of dimethylimidazole, and reacted with mechanical stirring for 10 min. After several washes with deionized water, the Fe_3_O_4_@ZIF-8 nanoparticles were collected by using magnetic attraction and dried in an oven for weighing convenience.

### DOX Loading and Release

Five mg of Fe_3_O_4_@ZIF-8 were dissolved in a 0.4 mg/mL DOX solution. After overnight shaking, the sediment was captured using magnetic attraction and underwent repeated washing with deionized water to obtain Fe_3_O_4_@ZIF-8/DOX. The drug loading efficiency was calculated by measuring the absorbance at 483 nm before and after drug loading. To assess the acid-responsive release capability of Fe_3_O_4_@ZIF-8/DOX, 5 mg of Fe_3_O_4_@ZIF-8/DOX were resuspended in 3 mL of PBS at varying pH levels (pH = 5.0, 6.5, or 7.2). The solution was placed in dialysis bags and immersed in centrifuge tubes containing 12 mL of PBS. Absorbance at 483 nm of the supernatant was assessed at different intervals (1, 2, 4, 8, 12, 24, 36, 48, 60, and 72 h) to calculate drug release amount based on the absorbance changes (n = 3).

### Cell culture

L15 medium (Solarbio) containing 10% (v/v) fetal bovine serum (FBS, Ausbian) and 1% (v/v) penicillin/streptomycin (P/S) antibiotics was utilized to culture SW620 cells. SW620 cancer cells were cultivated in an incubator with air at 37 °C.

### Cell Compatibility and Cytotoxicity

Cell compatibility of Fe_3_O_4_@ZIF-8 and cytotoxicity of Fe_3_O_4_@ZIF-8/DOX were both assessed using CCK-8 assay (n = 4). SW620 cells were plated in a 96-well plate and allowed to incubate overnight. Solutions of Fe_3_O_4_@ZIF-8 at various concentrations (1, 25, 50, 100, 200, 400, 800 μg/mL) were prepared by resuspending them in L15 medium, which were subsequently incubated with the SW620 cells. After 48 h, the cell viability was quantified using the CCK-8 assay. Additionally, Fe_3_O_4_@ZIF-8, DOX, and Fe_3_O_4_@ZIF-8/DOX were resuspended in L15 medium and incubated with overnight-cultured SW620 cells and NCM460 cells. After 48 h, cell cytotoxicity was quantified by using the CCK-8 assay.

### In Vitro Drug Delivery of Fe_3_O_4_@ZIF-8

SW620 cells were plated in a confocal dish and allowed to incubate overnight. FITC-labeled Fe_3_O_4_@ZIF-8 were resuspended in L15 medium to prepare a 100 μg/mL solution, which was then incubated with SW620 cells. After a 12 h incubation period, the nanoparticles were extracted, and the cells were subsequently immobilized with 4% paraformaldehyde for a duration of 30 min. Following fixation, the cells were stained with Actin-Tracker Red-555 and DAPI for the cell cytoskeleton and nucleus, respectively. Lysosome staining was performed by incubating SW620 cells with 100 μg/mL FITC-labeled Fe_3_O_4_@ZIF-8 for 4 h, followed by staining with Lyso Tracker (Life Technologies). After the excess staining solution was washed away, laser confocal microscopy was used for observation.

### Animal Model

Male Balb/c nude mice (6 weeks old) were sourced from the Hangzhou Medical College Experimental Animal Center. The experimental protocol was granted approval by the Institutional Animal Care and Use Committee of Zhejiang University, and the Institutional Animal Care and Use Committee approval number is ZJU20230397. All mice were accommodated in a specific pathogen-free (SPF) environment and were randomly allocated into five groups (n = 5). All procedures related to animal experimentation adhered to the established protocols of the Laboratory Animal Center at Zhejiang University.

### In Vivo Tumor Therapy

Subcutaneous injections of 6 × 10^5^ SW620 cancer cells were administered into the upper back area of male nude mice aged 6 weeks.^32^ Once the tumors reached an approximate volume of 50 mm^3^, the mice were assigned randomly to one of five groups (n = 5) for different drug treatments. The five groups were as follows: 1) Control (PBS), 2) Fe_3_O_4_@ZIF-8+Magnet, 3) free DOX, 4) Fe_3_O_4_@ZIF-8/DOX, and 5) Fe_3_O_4_@ZIF-8/DOX+Magnet. An external magnetic field was applied by securing a magnet block to the tumor site of the mice immediately after drug injection and removing it after 1 h. The size parameters of the magnet block are 10 mm (diameter) × 1.5 mm (thickness) in a circular shape. Tumor size and the weight of each mouse were documented every 3 days. Tumor tissues were collected and prepared into sections for H&E, TUNEL and *K*_i_-67 staining to analyze tumor cell proliferation and apoptosis.

### In Vivo Imaging

Free Rhodamine B (RhB) and Fe_3_O_4_@ZIF-8/RhB nanoparticles were injected into the tail veins of SW620 tumor-bearing mice separately. The loading method of RhB is the same as that of DOX, with a uniform concentration of RhB at 1 mg/mL. Immediately after injection, a magnet block was fixed on the tumor site of the mouse to apply a magnetic field for 1 h. Fluorescence images were taken at 0.5, 1, 4, and 12 h post injection, respectively. At 12 h postinjection, major organs (heart, liver, spleen, lung, kidney) and tumors were isolated to obtain fluorescence images and conduct fluorescence analysis (n = 3).

### Cardiotoxicity Assessment

M-mode echocardiographic images were collected and analyzed from three groups of mice including Control (PBS), free DOX, and Fe_3_O_4_@ZIF-8/DOX+Magnet group after 19 days of treatment (n = 5). Serum samples from the mice were used to measure the CK-MB and LDH1 levels. Heart tissues of nude mice bearing SW620 tumors in three groups were collected for section preparation. Tissue sections were subjected to TUNEL and Masson staining to observe whether there was any histological damage.

### Hemolysis Assay

One mL of blood from the normal nude mice was collected in an anticoagulant tube and centrifuged at 1000 g for 5 min to isolate the red blood cells. Afterward, these red blood cells were thoroughly washed with PBS through multiple cycles, until the supernatant became clear. Then, the red blood cell precipitate was resuspended in 5 mL of PBS. 300 μL of the aforementioned red blood cell solution was taken to mix with 700 μL of H_2_O or Fe_3_O_4_@ZIF-8 nanoparticles at different concentrations (6000, 3000, 1500, 800, 400, 200, 100, and 50 μg/mL). The blend was placed in a 37 °C incubator for 4 h, and then it was subjected to centrifugation at 6000 rpm for 10 min. 100 μL of the cleared supernatant was dispensed into a 96-well plate, and the optical density at 540 nm was documented (n = 6). The hemolysis percentage was determined using the following equation below: Hemolysis (%) = (Ab sample - Ab PBS)/(Ab H_2_O – Ab PBS) × 100%, where Ab stands for absorbance at 540 nm

### Histological Analysis

Heart, tumor tissues and primary organs (heart, liver, spleen, lung, kidney and brain) were fixed with in 4% tissue cell fixative and then embedded in paraffin. These paraffin-embedded tissues were sliced into 4 μm sections and stained with hematoxylin and eosin (H&E) for histopathological assessment. Fibrosis was evaluated using Masson trichrome staining, apoptosis was measured by TUNEL assay, and proliferation was assessed through Ki67 staining. ImageJ software was utilized for semiquantitative analysis.

### Blood Routine and Biochemical Analysis

Blood samples of SW620 tumor-bearing nude mice in all groups were collected for analysis (n = 5). A portion of the blood was gathered in anticoagulant tubes to undergo routine blood analysis. The remaining blood was allowed to clot, and after centrifugation, the serum was collected. The collected serum samples were used for biochemical analysis.

### Statistical Analysis

Data were plotted with standard error of mean (SEM). When the data satisfied homogeneity of variance and normal distribution, their significance was evaluated using a One-way ANOVA test. When one of these two criteria was not satisfied, the significance was evaluated using Kruskal–Wallis test. Significance for these statistical tests was defined at * *p* < 0.05, ** *p* < 0.01, *** *p* < 0.001.

## Supporting Information Available

Additional experimental data, including in vivo images and quantitative fluorescence analysis; hemolysis assay; H&E staining analysis of mice organs; routine blood tests; and biochemical blood analysis (PDF)
