# Supplementary material for: Precision Treatment of Colon Cancer Using Doxorubicin-Loaded Metal–Organic-Framework-Coated Magnetic Nanoparticles
Source: ACS Appl Mater Interfaces. 2024 Sep 3;16(37):49003–12. doi: 10.1021/acsami.4c08602 (PMC11420861; doi:10.1021/acsami.4c08602)
Supplement: Supplementary file 1 — am4c08602_si_001.pdf [file am4c08602_si_001.pdf]

Supporting Information

Precision Treatment of Colon Cancer Using Doxorubicin-Loaded Metal-Organic-Framework-Coated Magnetic Nanoparticles

*Honglin Jiang,[1] Qing Bao,[†1] Tao Yang,[1] Mingying Yang,[2] Chuanbin Mao[*3]*

[1]College of Materials Science & Engineering, Zhejiang University, Hangzhou 310027 Zhejiang, China

[2]Key Laboratory of Silkworm and Bee Resource Utilization and Innovation of Zhejiang Province, Institute of Applied Bioresource Research, College of Animal Science, Zhejiang University, Hangzhou 310058 Zhejiang, China

[3]Department of Biomedical Engineering, The Chinese University of Hong Kong, Shatin, Hong Kong SAR, China

*Correspondence should be addressed to C. Mao (cmao@cuhk.edu.hk)

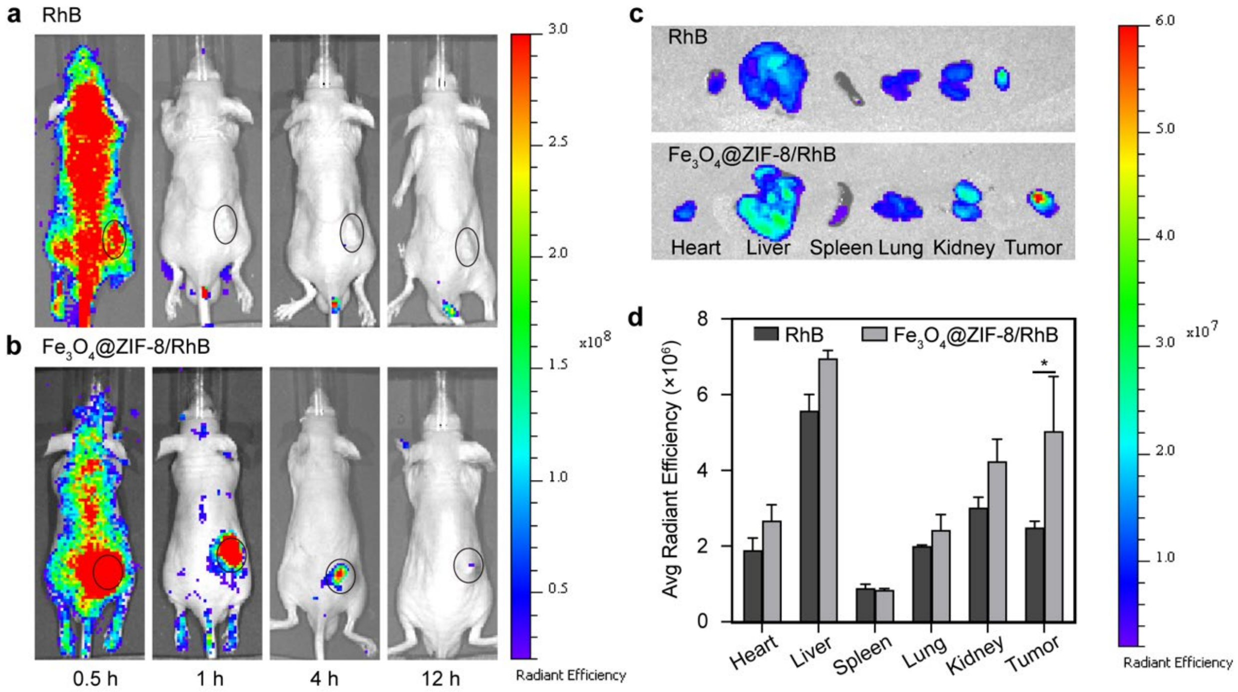

Figure S1. In vivo imaging of SW620 tumor-bearing mice. In vivo images taken at 0.5, 1, 4 and 12 h time point after administration of a) free RhB and b) Fe_3O_4@ZIF-8/RhB nanoparticles. c) Fluorescence images and d) quantitative fluorescence analysis of organs and tumors in SW620 tumor-bearing mice at 12 h post-injection of free RhB and Fe_3O_4@ZIF-8/RhB nanoparticles. (n=3). * $p<0.05$, ** $p<0.01$, *** $p<0.001$. The ovals in a and b indicate the tumor sites.

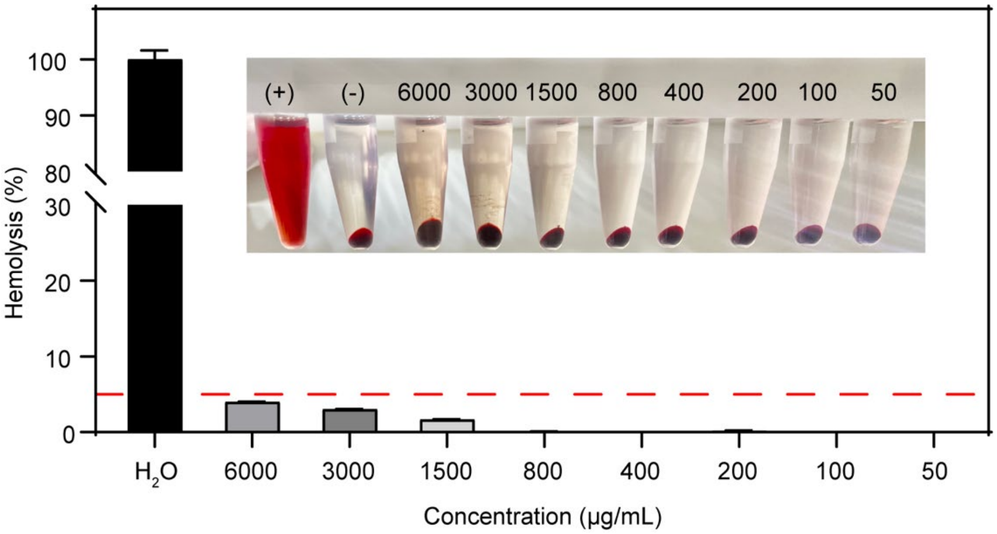

Figure S2. Hemolysis assay of Fe_3O_4@ZIF-8 nanoparticles at different concentrations. The red line represents a 5% hemolysis rate. (n=6).

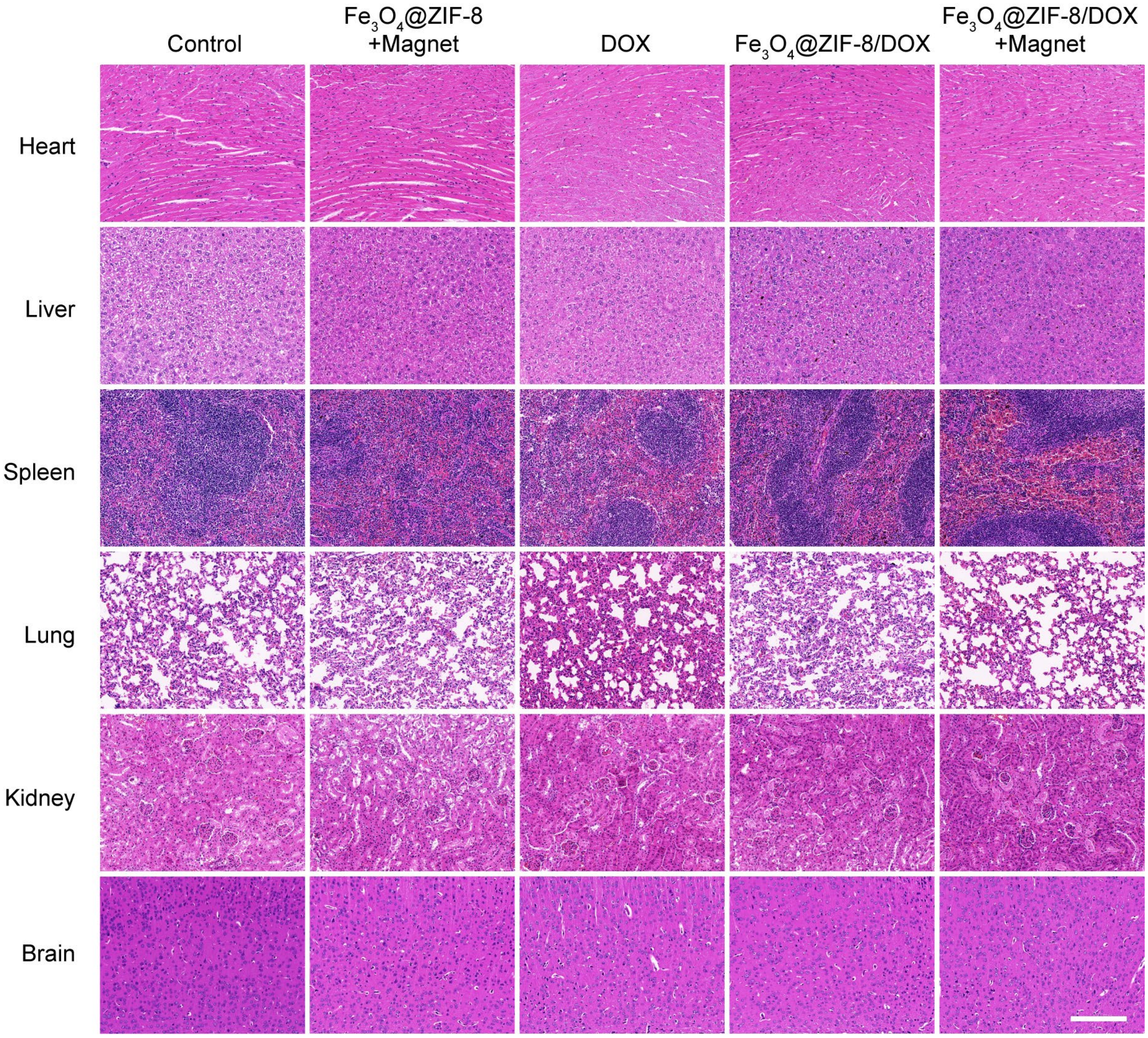

Figure S3. Hematoxylin and eosin staining of primary organs (heart, liver, spleen, lung, kidney, brain) excised from five sets of nude mice. The scale bar is 200 μm.

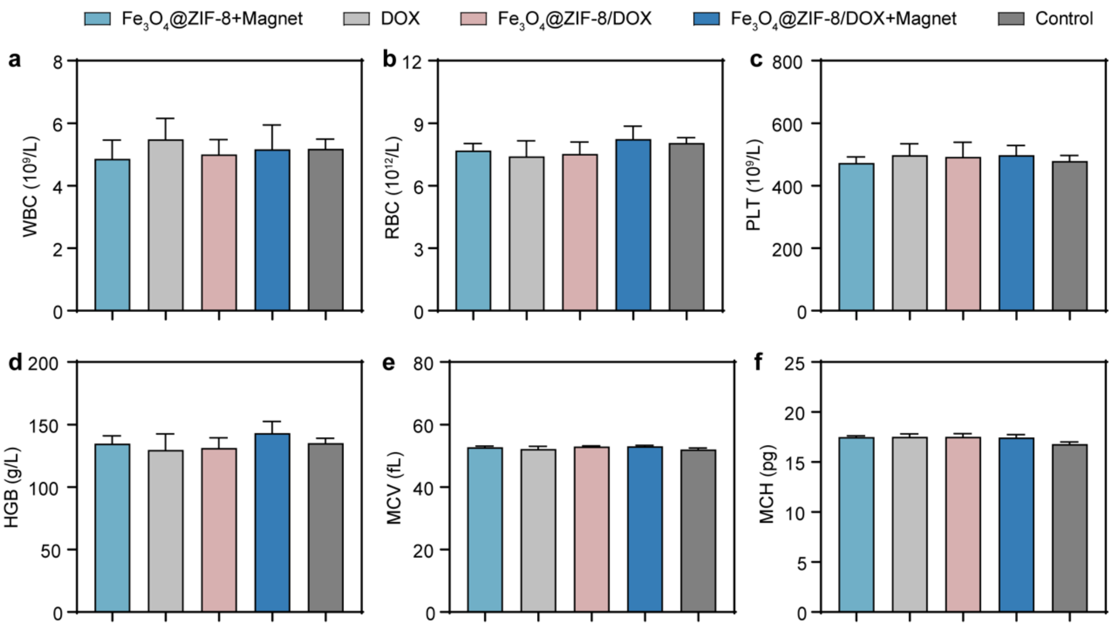

Figure S4. Routine blood tests for SW620 tumor-bearing nude mice (n=5), including: a) white blood cells (WBC), b) red blood cells (RBC), c) platelets (PLT), d) hemoglobin (HGB), e) mean corpuscular volume (MCV), f) mean corpuscular hemoglobin (MCH).

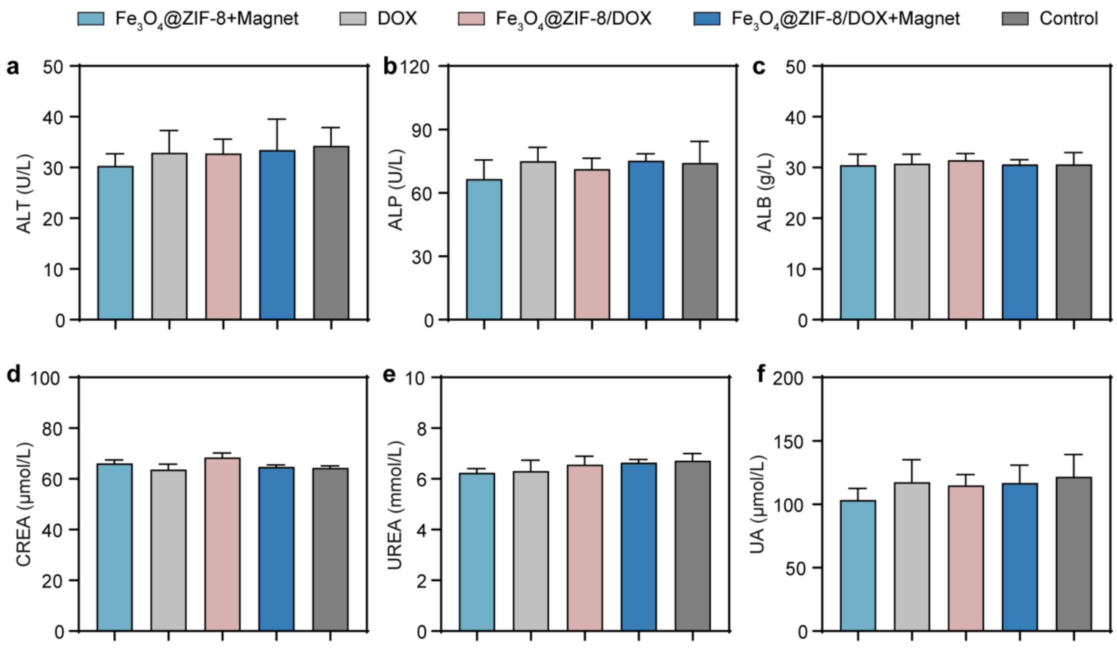

Figure S5. Biochemical assessment of liver and kidney function in SW620 tumor-bearing nude mice (n=5), including: a) alanine aminotransferase (ALT), b) alkaline phosphatase (ALP), c) albumin (ALB), d) creatinine (CREA), e) urea (UREA), f) uric acid (UA).
